# Self-healing hydrogels loaded with Spatholobi Caulis alleviate disc degeneration by promoting autophagy in nucelus pulposus

**DOI:** 10.1016/j.mtbio.2024.101323

**Published:** 2024-11-08

**Authors:** Shenghao Cai, Rui Ding, Hongjun Zhang, Qirui Chen, Fen Yu, Yong Xia, Qi Chen, Xinxin Miao, Bin Zhou, Jiahui Chen, Le Liao, Xigao Cheng, Xiaoling Fu

**Affiliations:** a Department of Orthopedics, The Second Affiliated Hospital, Jiangxi Medical College, Nanchang University, Nanchang, Jiangxi, China; b Institute of Orthopedics of Jiangxi Province, Nanchang, Jiangxi, China; c Affiliated Rehabilitation Hospital of Nanchang University, China; d School of Materials Science and Engineering, East China Jiaotong University, Nanchang, Jiangxi, China; e Jiangxi Medical College, Nanchang University, Nanchang, Jiangxi, China

**Keywords:** Autophagy, Spatholobi caulis, Self-healing hydrogel, Disc degeneration

## Abstract

Intervertebral disc degeneration (IDD) is a common degenerative disease of the spine that has a significant impact on both society and human health. Many studies have confirmed that there is a close relationship between IDD and senescence and apoptosis, and autophagy can combat apoptosis and senescence. Spatholobi caulis (SC) is an herb that contains various active compounds that are effective in tissue repair and regeneration, but it has not been explored in field of IDD. In this study, it was first found that SC can boost autophagy and reduce the apoptosis and senescence of Nucleus pulposus cell (NPCs). However, our animal studies revealed limited absorption of SC. To improve the bioavailability and efficacy of SC, we developed a hydrogel incorporating quaternary ammonium chitosan (QCS) and oxidized starch (OST) as carriers for SC. The QCS-OST/SC hydrogel exhibits excellent compatibility with cells, can be easily injected, and can release SC durably. At the cellular level, the QCS-OST/SC hydrogel enhances cell viability, initiates autophagy and release of the extracellular matrix (ECM), and inhibits cellular senescence and apoptosis. The injection of the QCS-OST/SC hydrogel via microneedles (MNs) into discs had successfully diminished disc degeneration in rats, which shows that this hydrogel has broad potential in the treatment of IDD.

## Graphical abstract

**Figure:**
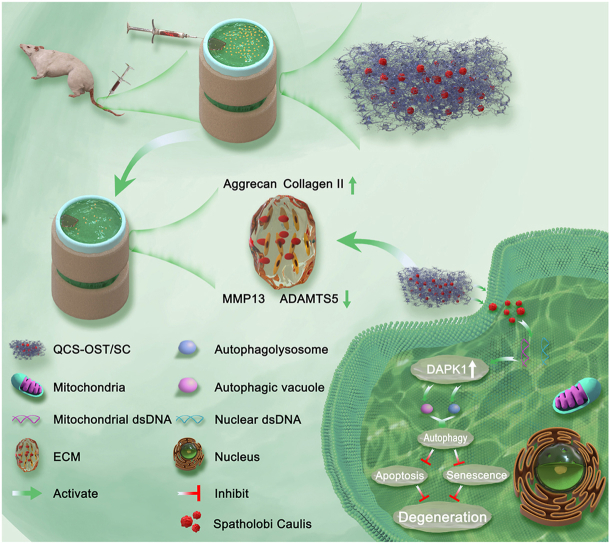

## 1 Introduction

Intervertebral Disc Degeneration (IDD) is a prevalent age-related condition that can result in significant disability and has become a leading public health issue globally [1].

Current treatment modalities for IDD encompass growth factor therapy, cell therapy, and gene therapy [2]. Growth factor therapy involves the topical application of bioactive molecules to the disc, which interact with specific cell surface receptors to modulate cell proliferation, differentiation, and ECM synthesis. Thompson et al. demonstrated the first successful exogenous administration of a growth factor (TGF-β1) in an animal model, which led to an upregulation of proteoglycan synthesis in nucleus pulposus cells [3]. Given the chronic nature of IDD, repeated injections or combinations of growth factors may be necessary due to their short half-life and instability when directly injected into the disc. The primary objective of cell therapy is to replenish deceased and apoptotic cells by injecting stem cells, which can further differentiate and replace necrotic and apoptotic nucleus pulposus cells during disc degeneration, thereby minimizing cell mortality within the disc. Richardson and Hoyland have reported on the potential use of various adult stem cells for disc regeneration [4]. However, cell therapy has certain limitations, and maintaining the viability of the injected cells and preventing their leakage remain significant challenges to be addressed. Gene therapy employs vectors carrying specific genes to transfer genetic information into the host cell DNA, modulating gene expression levels [5]. Nevertheless, the immune responses and mutagenicity associated with gene therapy are still uncontrollable, and the therapeutic targets are relatively singular. Therefore, there is an imperative need to identify new therapeutic strategies for intervertebral disc degeneration that offer a broader range of targets, improved stability, and superior safety profiles.

Recent studies have suggested it has a shift toward a younger patient population, resulting in a significant increase in cases [6]. The disc is consisted of the NP and annulus fibrosus (AF). NP is the main functional component of the disc, which allows disc to effectively withstand mechanical stresses. The annulus fibrosus creates a supportive ring structure surrounding the NP [7]. NPCs which make up the NP and are responsible for producing the ECM, are vital elements in the intervertebral disc [8]. The key components of the ECM include collagen and proteoglycans, which are critical for maintaining disc integrity. Notably, excessive apoptosis and senescence of NPCs have a significant impact on their normal function, which makes them to be potential targets for treating IDD [9]. Autophagy is a cellular process in which dysfunctional organelles and proteins are disrupted to maintain cell health and improve resistance to stress [10]. Autophagy is closely related to the development of IDD and is crucial for regulating NPCs apoptosis and senescence [11]. Various degenerative conditions, such as osteoarthritis [12], age-related macular degeneration [12], and Alzheimer's disease [13], have been connected to abnormal autophagy. Recent research indicates that autophagy serves as a protective mechanism for NPCs, defending against apoptosis and senescence [9].

Spatholobi Caulis, a Chinese herbal medicine known for its diverse bioactive compounds, has been extensively studied [14]. The effects of SC include angiogenesis promotion [15], antiviral [16] and antioxidant properties [17], and anti-inflammatory [18] and antiapoptotic effects [19]. It has shown promise in treating osteosarcoma [20], obesity [21], and ischemic stroke [19], but its potential for treating IDD remains unexplored. Our research suggested that SC may be an effective treatment for lumbar disc degeneration. While SC has low toxicity, traditional high-frequency perfusion may be toxic and have limited bioavailability [22]. Direct injection into the intervertebral disc could result in rapid clearance and reduced efficacy in treating IDD. Thus, a delivery system is needed to enhance the therapeutic effects of SC in IDD treatment.

Starch has the advantages of a wide range of sources, low cost and good biocompatibility and is widely used in biomedical fields [23]. However, due to the lack of ionizable functional groups, natural starch has low solubility, weak adsorption, and poor mechanical properties, which limits its practical application as a high quality delivery materials to a certain extent [24].Therefore, it is necessary to modify starch by chemical modification, physical modification, enzyme modification and other methods to prepare better starch-based materials. Although starch-based hydrogels have good biocompatibility and biodegradability, their homogeneity and mechanical properties are poor, which also affects their application [25]. To improve the performance of starch hydrogels, other natural polysaccharide polymers, such as cellulose, chitosan, and gelatin, can be used to form composite hydrogels with starch. Among them, chitosan not only has good biocompatibility and biodegradability but can also increase the crosslinking density of starch-based hydrogels and enhance their mechanical properties [26]. Therefore, chitosan can be utilized to augment the mechanical strength of starch-based hydrogels, and improve the application value of slow drug release and tissue engineering [27].

**Scheme 1:**
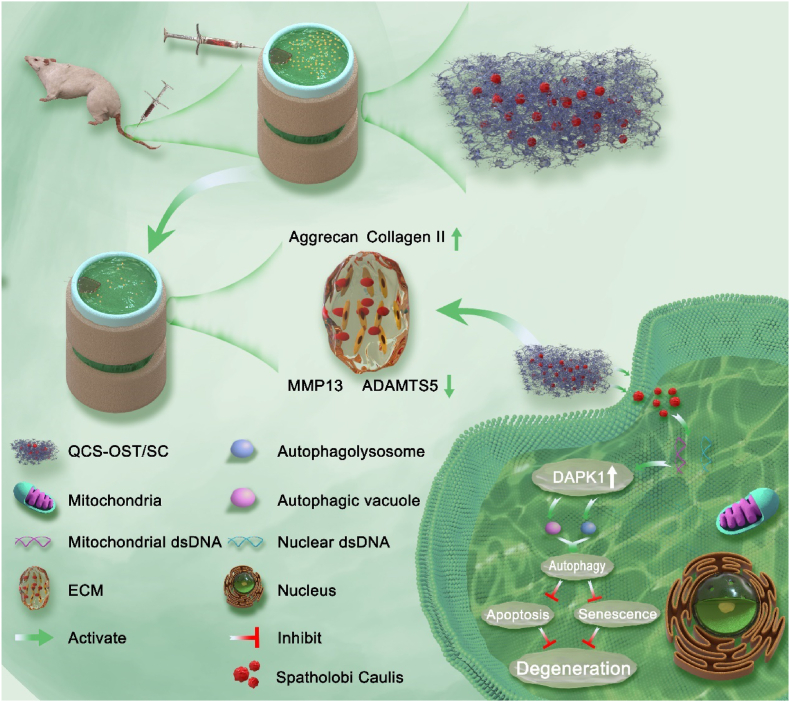
Therapy for intervertebral disc degeneration employing an injectable, self-mending hydrogel as a delivery system. After the injection of QCS-OST/SC hydrogel into the degenerated intervertebral discs of rats, the SC particles encapsulated within the QCS-OST hydrogel are slowly released. By modulating the high expression of DAPK1 protein, the number of autophagosomes and autolysosomes in nucleus pulposus cells significantly increases, inhibiting apoptosis and senescence of these cells, and promoting the release of extracellular matrix. This significantly slows down the progression of intervertebral disc degeneration.

Based on the above, in this study, a multifunctional injectable hydrogel was designed and prepared by using the natural high molecular weight polymers starch and chitosan as raw materials (Scheme 1). First, starch and chitosan were converted into oxidized starch (OST) and quaternary ammonium chitosan (OCS), respectively, by modification technology, and the QCS-OST hydrogel was successfully synthesized by physical crosslinking and used as the transfer substrate of SC. The composite hydrogel not only has good antibacterial, anti-inflammatory and antioxidant properties but can also achieve the slow release of SC for many days. Furthermore, this 3D porous SC-chitosan hydrogel system (QCS-OST/SC hydrogel) was evaluated in a rat model of intervertebral disc degeneration, demonstrating its ability to stimulate autophagy within the NP and to suppress apoptosis and senescence of the disc cells. The application of the QCS-OST/SC hydrogel is positioning itself as a favorable tactic for IDD management, with encouraging prospects for its use in therapy.

## 2 Experimental results

### 2.1 SC can reduce the apoptosis and senescence of NPCs

**Fig. 1:**
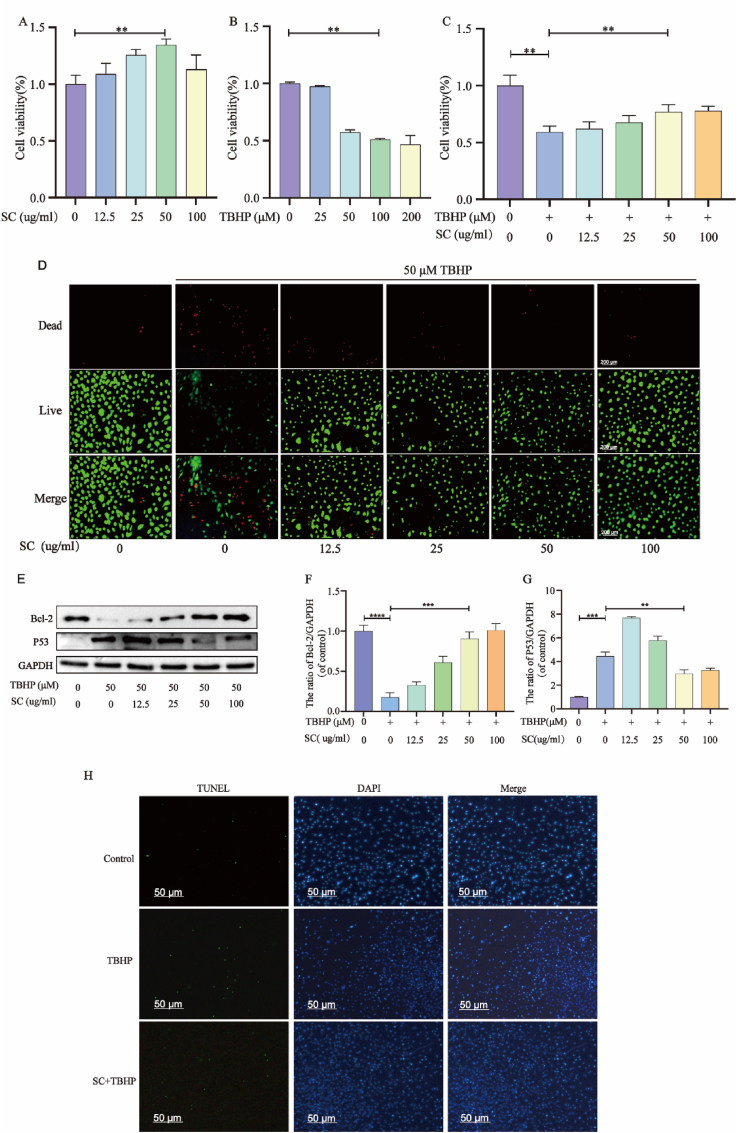
**SC treatment can reduce the apoptosis and senescence of NPCs. (A)** CCK-8 assay depicts NPC viability following 24-h SC exposure across a concentration gradient. **(B)** NPCs response to 24-h TBHP treatment at varying concentrations as indicated by CCK-8. **(C)** CCK-8 reflects NPC viability post-SC pre-treatment and subsequent TBHP challenge. **(D)** Live-dead assay visualizes NPCs status after SC pre-conditioning and TBHP exposure (scale bar = 200 μm). **(E**–**G)** Bcl-2 and P53 protein levels in NPCs under TBHP exposure, modulated by SC co-treatment. **(H**–**I)** TUNEL assay quantifies apoptotic NPCs following TBHP treatment, with or without SC pre-treatment (scale bar = 50 μm).The data in the figure represent mean ± S.D. Significant differences between the treatment and control groups are expressed as ∗∗∗P < 0.001, ∗∗p < 0.01, ∗p < 0.05, n = 3.

SC (Fig. S1) increased the viability of NPCs at a concentration of 50 μg/mL for 24 h (Fig. 1A). In contrast, exposure to TBHP led to a decrease in cell viability that was dose dependent (Fig. 1B). SC had a significant protective effect on TBHP-induced apoptosis (Fig. 1C). When treated with 50 μM TBHP, the number of dead cells increased significantly, while SC shielded these cells from TBHP-triggered decreased viability (Fig. 1D, Fig. S2). WB revealed that TBHP (50 μM) decreased the levels of the antiapoptotic protein Bcl-2 and increased the levels of the senescence marker P53. SC preconditioning reversed the expression of Bcl-2 and inhibited the expression of P53 in NPCs post-TBHP treatment (Fig. 1E–G). These results were corroborated by TUNEL staining and flow cytometry (Fig. 1H, Figure S3-4).

### 2.2 SC activates DAPK1 and induces autophagy in NPCs

**Fig. 2:**
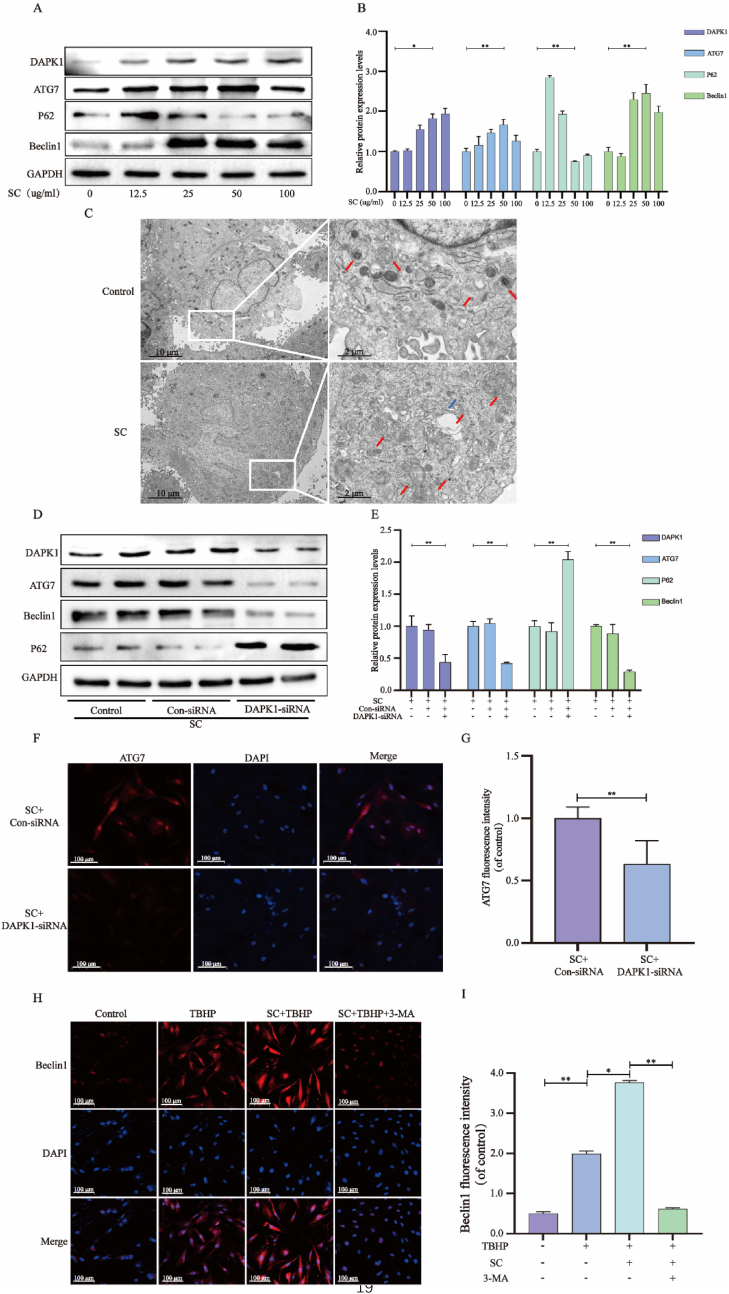
**SC activates DAPK1 and induces autophagy in NPCs.** NPCs were incubated with 0, 12.5, 25, 50, or 100 μg/mL SC for 24 h. (**A-B**) Protein content of DAPK1, ATG7, P62, and Beclin-1 in treated NPCs as described above. (**C**) Autophagosomes and autopolysosomes (red arrow autophagosomes, blue arrow autopolysosomes) in NPCs were detected by transmission electron microscopy (scale bar = 10 μm and 2 μm). After 24 h of SC pretreatment, NPCs were treated with 100 μM DAPK1-siRNA, and (**D-E**) the protein contents of DAPK1, ATG7, P62, and Beclin-1 in the treated NPCs as described above. (**F-G**) Immunofluorescence of DAPK1 of treated NPCs as described above (scale bar = 100 μm). (**H-I**) The immunofluorescence of Beclin-1 in NPCs was treated with TBHP, SC + TBHP and SC + TBHP + 3-MA, respectively (scale bar = 100 μm). The data in the figure represent mean ± S.D. Significant differences between the treatment and control groups are expressed as ∗∗p < 0.01, ∗p < 0.05, n = 3.

We used WB measurements of ATG7, Beclin1, P62, and DAPK1/GAPDH ratios as markers of autophagy. Treatment of NPCs with SC for 24 h resulted in increase in DAPK1, ATG7 and Beclin-1 expression, while P62 levels decreased at 50 μg/mL and 100 μg/mL SC (Fig. 2A and B). Furthermore, changes in the presence of autophagosomes and autophagolysosomes were detected through the use of transmission electron microscopy, with SC-treated cells (50 μg/mL) displaying greater numbers of autophagosomes and autophagolysosomes than control cells (Fig. 2C). By transfecting cells with DAPK1-siRNA before SC treatment, we noticed a decrease in ATG7 and Beclin-1 levels and an increase in P62 levels, confirming the involvement of DAPK1 in SC-induced autophagy activation (Fig. 2D and E). Moreover, the immunofluorescence results showed that after DAPK1 was successfully knocked out, ATG7 also decreased (Figure F–G). Pretreatment with the autophagy inhibitor 3-MA blocked SC-induced autophagy in NPCs (Figure H–I, Fig. S5).

### 2.3 SC neutralizes the effects of TBHP on promoting senescence and apoptosis in NPCs by inducing autophagy

**Fig. 3:**
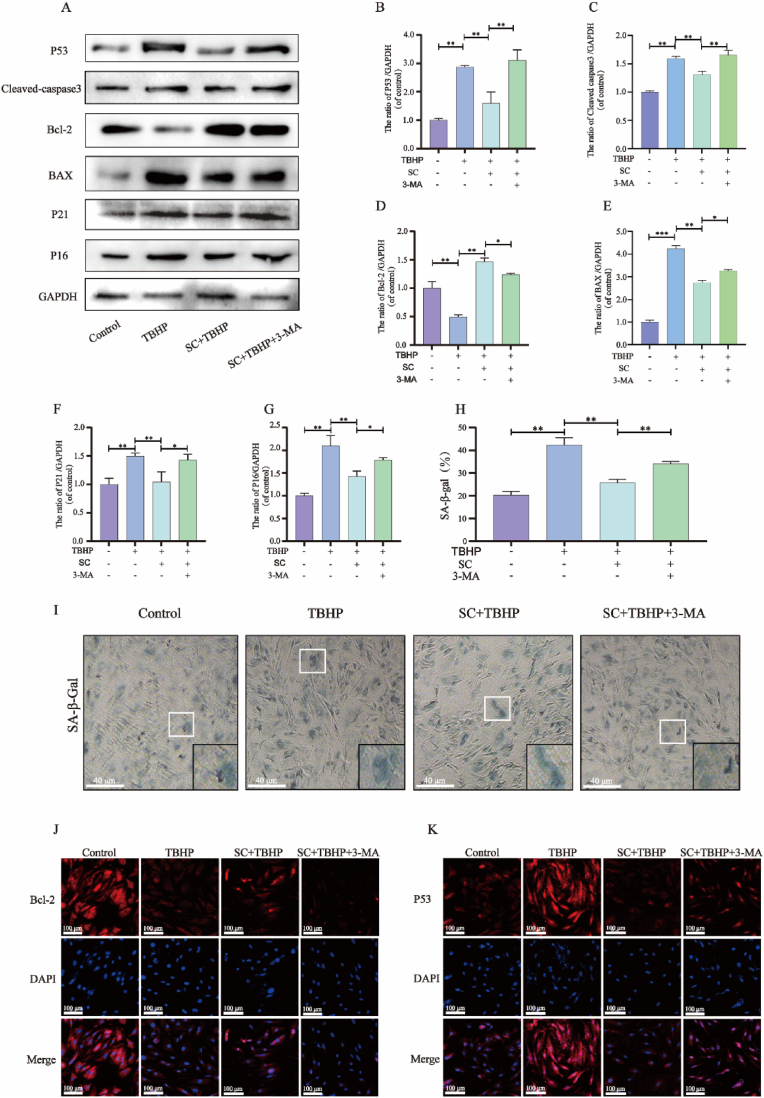
**SC neutralizes the effects of TBHP on promoting senescence and apoptosis in NPCs by inducing autophagy.** NPCs were untreated (DMEM 10 % FBS), treated with TBHP alone, treated with SC (50 μg/mL) and TBHP, or treated with TBHP and SC (50 μg/mL) combined with 3-MA (10 mM). (**A-I**) Protein content of Caspase3, Bcl-2, BAX, P53, P21 and P16 in treated NPCs as described above. (I) The NPCs were examined for senescence using SA-β-gal staining according to the previously outlined methodology (scale bar = 40 μm). (**J-K**) Immunofluorescence assays of Bcl-2 and P53 were performed in NPCs treated as above (scale bar = 100 μm). The data in the figure represent mean ± S.D. Significant differences between the treatment and control groups are expressed as ∗∗p < 0.01, ∗p < 0.05, n = 3.

To explore the impact of autophagy on the protective effect of SC-induced apoptosis in NPCs, pretreatment with the autophagy inhibitor 3-MA was administered to the cells. WB analysis revealed a notable increase in apoptosis in cells exposed to TBHP and 3-MA, while a decrease in apoptosis was observed in cells treated with SC. TBHP treatment resulted in a decrease in the protein level of Bcl-2 and an increase in the protein levels of Bax and Cleavedcaspase3, whereas SC alleviated TBHP-induced apoptosis. Compared with those in the control group, the TBHP group displayed increased levels of the Bax and Cleaved-caspase3 proteins and decreased levels of the Bcl-2 protein. However, the impact of SC was nullified by 3-MA (Fig. 3A,C-E). Following exposure to TBHP, senescent NPCs exhibiting positivity for SA-β-gal and heightened levels of P53, P21, and P16 were observed, which were notably suppressed by SC (Fig. 3I). On the other hand, the inhibition of autophagy by 3-MA weakened the anti-senescence effect induced by SC. Western blot data illustrated that SC treatment reduced the expression of senescence-related proteins, including P53, P21, and P16, while TBHP increased their expression (Fig. 3A, B, F-H). These observations were further supported by immunofluorescence staining, which indicated that SC hindered TBHP-induced apoptosis and senescence in NPCs through autophagy (Figure J–K).

### 2.4 SC regulates the expression of metabolism-related genes through autophagy

**Fig. 4:**
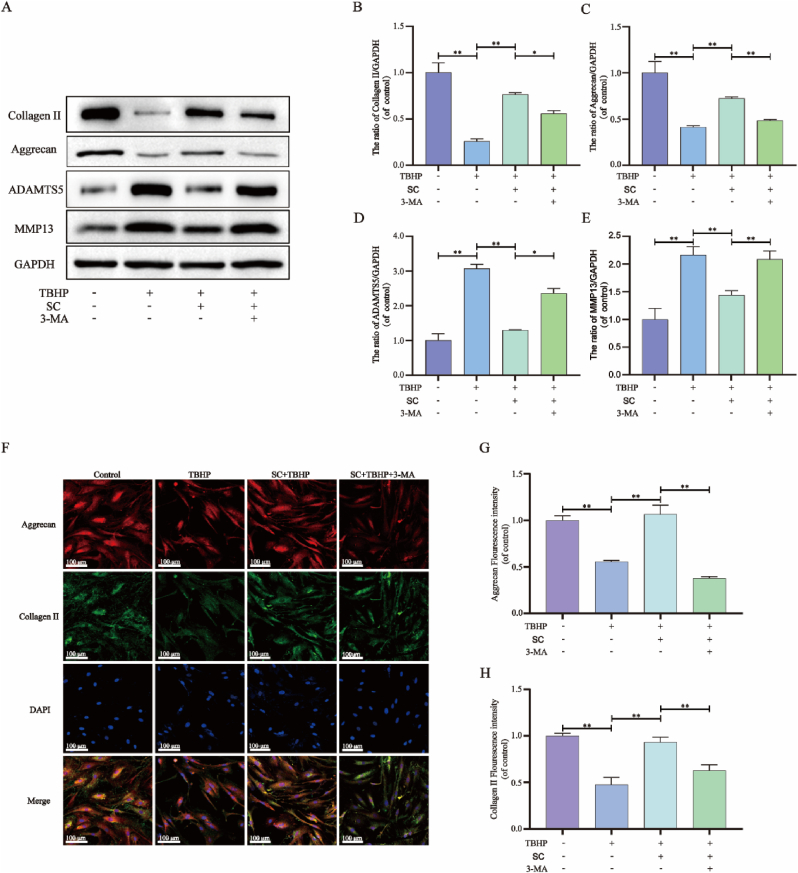
**SC regulates the expression of metabolism-related genes through autophagy.** NPCs were untreated (DMEM 10 % FBS), treated with TBHP alone, treated with SC (50 μg/mL) and TBHP, or treated with TBHP and SC (50 μg/mL) combined with 3-MA (10 mM). (**A-E**) Protein content of Collagen II, Aggrecan, ADAMTS5 and MMP13 in treated NPCs as described above. (**F-H**) Immunofluorescence assays of Aggrecan and Collagen II were performed in NPCs treated as above (scale bar = 100 μm). The data in the figure represent mean ± S.D. Significant differences between the treatment and control groups are expressed as ∗∗p < 0.01, ∗p < 0.05, n = 3.

To assess the metabolic activity of NPCs more effectively, we investigated the primary extracellular matrix (ECM)-producing proteins Collagen II and Aggrecan, as well as the ECM-binding proteins MMP13 and ADAMTS5. After the indicated treatments, we analyzed the expression levels of these genes and proteins using WB and immunofluorescence. Exposure to TBHP resulted in a notable reduction in Collagen II and Aggrecan protein levels, while leading to an increase in MMP13 and ADAMTS5 protein levels. Intriguingly, the impacts of TBHP treatment were reversed by the activation of autophagy through SC, and this finding was further reinforced by the use of the autophagy inhibitor 3-MA (Fig. 4A–E). Immunofluorescence analysis of the Collagen II and Aggrecan protein levels validated the WB findings, indicating that SC could increase the levels of the Collagen II and Aggrecan proteins, and conversely, 3-MA could counter the increase in the expression of degeneration-associated genes induced by SC (Fig. 4F–H). Overall, our findings suggest an increase in Collagen II and Aggrecan expression in conjunction with a decrease in MMP13 and ADAMTS5 expression.

### 2.5 Synthesis and characterization of hydrogels loaded with SC

**Fig. 5:**
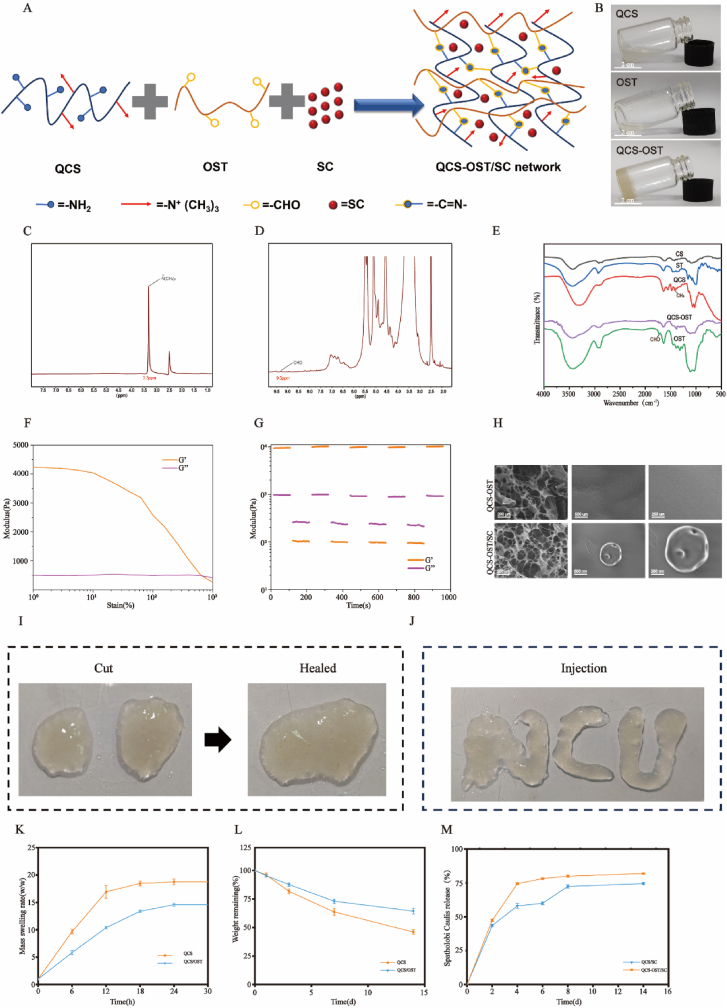
**Synthesis and characterization of SC-loaded hydrogel. (A)** Illustrative representation of the preparation process and molecular architecture of the QCS-OST hydrogel. **(B)** A step illustrating the combination and mixing of QCS and OST on ice to achieve the formation of the QCS-OST hydrogel (scale bar = 2 cm). **(C**–**D)** Proton Nuclear Magnetic Resonance (^1^H NMR) spectral analysis of the quaternized chitosan and the modified starch, showcasing their characteristic peaks. **(E)** Fourier Transform Infrared (FT-IR) spectroscopy profiles of the chitosan, quaternized chitosan, starch, oxidized starch, and the composite QCS/OST hydrogel, highlighting their chemical compositions. **(F)** Assessment of the viscoelastic properties and the point of strain transition for the QCS-OST hydrogels. **(G)** Evaluation of the self-healing efficiency of the QCS-OST hydrogels under a cyclic strain condition. **(H)** SEM micrographs showed that the porous structures of hydrogels and SC were distributed on the matrix of QCS-OST hydrogels (scale bar = 200 μm, 500 nm and 200 μm). **(I)** Images showing the macroscopic self-healing behavior of QCS-OST hydrogel. **(J)** QCS-OST hydrogel can be easily injected to write letters. **(K**–**L)** Swelling rate and weight retention rate of QCS-OST and QCS. (M) Cumulative release curve in SC hydrogel (n = 3).

The synthesis of QCS-OST hydrogels is achieved through the formation of a Schiff base linkage between o-toluidine (OST) and quaternized chitosan (QCS), as depicted (Fig. 5A). Initially, chitosan and soluble starch undergo modification through standard chemical processes. The hydrogel is then created by combining QCS and OST at a specific concentration ratio while maintaining the mixture on ice, as illustrated (Fig. 5B). The ^1^H NMR spectra of QCS and OST are displayed in Fig. 5C and D, respectively. The presence of a 3.3 ppm peak in the QCS spectrum confirmed the successful synthesis of QCS [28]. OST, the peak at approximately 9.3 ppm suggested the oxidation of the hydroxyl group to an aldehyde group [29]. The chemical structures of all the samples were further confirmed using FT-IR. A distinctive peak observed at 1479 cm^−1^ in the QCS spectrum [30], which was not present in the chitosan spectrum, confirms the successful bonding of GTMAC to chitosan. Likewise, the appearance of a peak at 1725 cm^−1^ in the OST spectrum, previously unseen in the soluble starch spectrum, signifies the presence of a distinctive aldehyde group in the modified starch [31], which was absent in the soluble starch sample indicates the existence of the characteristic aldehyde group in OST. Furthermore, the QCS-OST sample exhibits a peak at 1572 cm^−1^, corresponding to the stretching vibration of the C=N group resulting from the interaction between OST and QCS [32]. (Fig. 5E).

This study further examined the rheological properties, self-repair ability, and injectability of the hydrogels. The research outcomes demonstrated that the QCS-OST hydrogel possessed an energy storage modulus around 500 Pa, suggesting optimal mechanical attributes for intervertebral disc repair. The transition point, where the hydrogel shifted from solid to fluid state, was identified at a critical strain of about 645.4 %, marking the changeover between the storage and loss moduli of the hydrogel network (Fig. 5F). Quantitative self-healing property was assessed by rheology behavior testing. The QCS-OST samples were able to maintain a solid-state under minimal strain and transition to a liquid state under high strain. Cyclic rheological testing confirmed the exceptional self-repairing ability of the QCS-OST hydrogel (Fig. 5G). Upon visual inspection, damaged hydrogels could merge and mend themselves, underscoring the self-healing characteristics of the QCS-OST hydrogels (Fig. 5I). The injectability performance of the QCS-OST sample was shown in Fig. 5G. These self-repairing properties are essential for preventing the hydrogel network from breaking apart, thereby preventing mechanical stress imbalances and additional degenerative harm to the disc and neighboring endplate cartilage. SEM analysis was utilized to evaluate the morphologies of the hydrogels, which revealed porous structures present in both samples. The successful incorporation of SC into the hydrogel pores was evidenced by the circular shape of the nanostructure observed in the QCS-OST/SC sample. When the QCS/OST and QCS are placed in water, the change is less in volume of the QCS/OST, which is highly important for preventing nerve compression due to volume changes after injection (Fig. 5K). The degradation of the hydrogel was observed in PBS at 37 °C. The degradation rate of QCS on the first day was 96 ± 1.73 %, while that of QCS-OST was 95.3 ± 1.15 %, with no significant difference. On the seventh day, the degradation rate of QCS was 63.7 ± 2.08 %, while that of QCS-OST was 73.0 ± 2.0 %. This obvious difference may be due to the addition of OST increasing the structure density, which has a significant long-term impact on the degradation rate and release rate. Hydrogels with stable structures promote cell adhesion, proliferation and extracellular matrix production in vitro (Fig. 5L). More than 60 % of the SC encapsulated in QCS was released on the fourth day, whereas the QCS-OST hydrogel matrix loaded with SC demonstrated the accumulation and sustained release of SC for up to 8 days (Fig. 5M). This prolonged release mechanism ensures the sustained efficacy of SC in treating disc degeneration, ultimately reducing the number of injections.

### 2.6 The QCS-OST/SC hydrogel can regulate the level of autophagy, apoptosis and senescence in NPCs

**Fig. 6:**
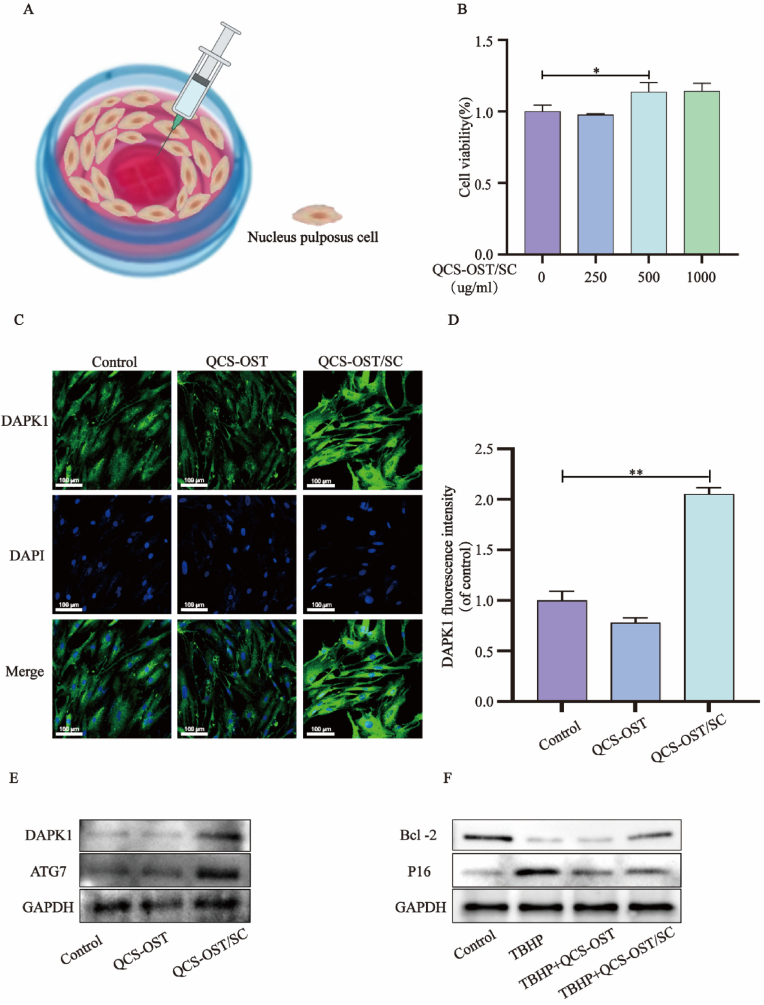
**QCS-OST/SC hydrogel can regulate the level of autophagy apoptosis and senescence of NPCs.** (**A**) Schematic illustration of NPCs co-cultured with hydrogel. Different experimental ingredients are injected into the non-contact culture chamber and the NP cells are inoculated on the bottom of petri dishes. (**B**) As mentioned above, CCK8 results were obtained after coculture of QCS-OST/SC hydrogel (containing 0, 250, 500 and 1000 μg/mL SC) and NPCs during 24 h. (**C)** Expression of DAPK1 in cells cocultured with PBS, QCS-OST and 500 μg/mL QCS-OST/SC groups for 24 h (scale bar = 100 μm). (**D)** Quantitative analysis of DAPK1 in NPCs (n = 3). (**E**) Expression of ATG7 and DAPK1 proteins in cells cultured with PBS, QCS-OST and QCS-OST/SC groups for 24 h. (**F**) Western blot images of Bcl-2 and P16 protein expression in treated NP cells from four independent experiments. The data in the figure represent mean ± S.D. Significant differences between the treatment and control groups are expressed as ∗∗p < 0.01, ∗p < 0.05, n = 3.

The QCS-OST/SC hydrogel was utilized in the Transwell chamber for the coculture of cells and drugs (Fig. 6A). The CCK-8 assay revealed that a concentration of 500 μg/mL QCS-OST/SC increased the viability of NPCs (Fig. 6B). Since 500 μg/mL SC of QCS-OST can effectively promote NPC activity, this dose was chosen for the following experiments. Immunofluorescence evaluation proved that 500 μg/mL QCS-OST/SC could enhance the expression of DAPK1 in NPCs (Figure C–D). Additionally, WB confirmed that QCS-OST/SC enhances the levels of ATG7 and DAPK1 while counteracting the reduction in Bcl-2 and increase in P16 induced by TBHP (Fig. 6E, F, Fig. S6). We were also surprised to find that QCS-OST hydrogel could inhibit the aging of NPCs to a certain extent. Therefore, at the cellular level, QCS-OST/SC effectively promoted cell viability and autophagy.

### 2.7 Sustained-release effect of QCS-OST/SC intervertebral disc injection

**Fig. 7:**
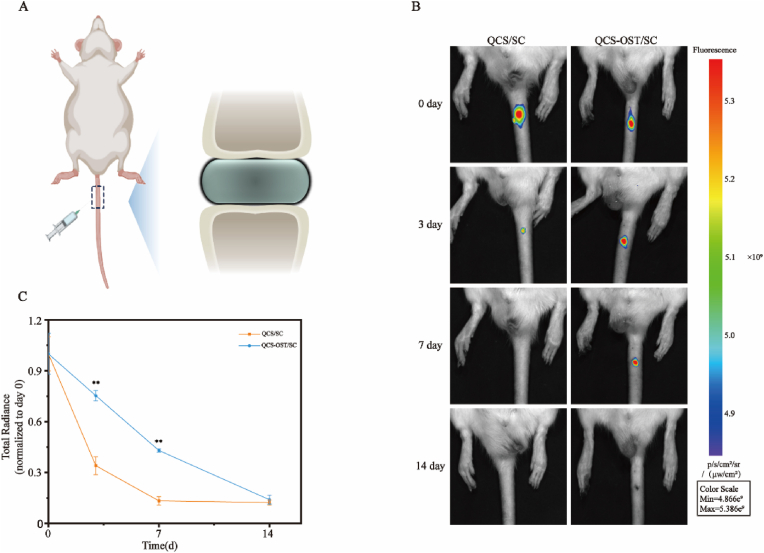
**Sustained-release effect of QCS-OST/SC intervertebral disc injection. (A)** Schematic representation of the injection process for Cy5-QCS/SC and Cy5-QCS-OST/SC hydrogels into the discal region. **(B)** Exemplary IVIS images documenting the evolution of fluorescence signal intensity at the disc sites over time in rats. The thermal maps (ranging from blue to red) correspond to a scale of fluorescence intensity, with blue indicating lower and red indicating higher intensities. **(C)** Graphical quantification of the fluorescence data extracted from the IVIS imaging of Cy5-QCS/SC and Cy5-QCS-OST/SC hydrogels. The data in the figure represent mean ± S.D. Significant differences between the treatment and control groups are expressed as ∗∗p < 0.01, n = 3.

To validate the durable release of SC, hydrogels of Cy5-QCS/SC and Cy5-OCS-OST/SC were produced through the labeling of CS with Cy5. These products were administered to the intervertebral discs of rats to monitor SC release behavior in vivo (Fig. 7A). After the administration of Cy5-QCS/SC, the fluorescence in the disc area was rapidly dispersed and eliminated on 3rd day. No visible fluorescence signal was detected on 7th day, consistent with in vitro results. Conversely, the Cy5-OCS-OST/SC hydrogel exhibited a persistent fluorescence signal on the 7th day, indicating a notably prolonged release duration in comparison to that of Cy5-QCS/SC (Fig. 7B and C). The increased release duration in vivo is thought to be due to the restricted distribution of SC within the hydrogel. These outcomes indicate the OCS-OST hydrogel can improve the sustained release capacity of SC, resulting in a continuous efficient concentration of SC within the intervertebral disc.

### 2.8 Animal experiments

**Fig. 8:**
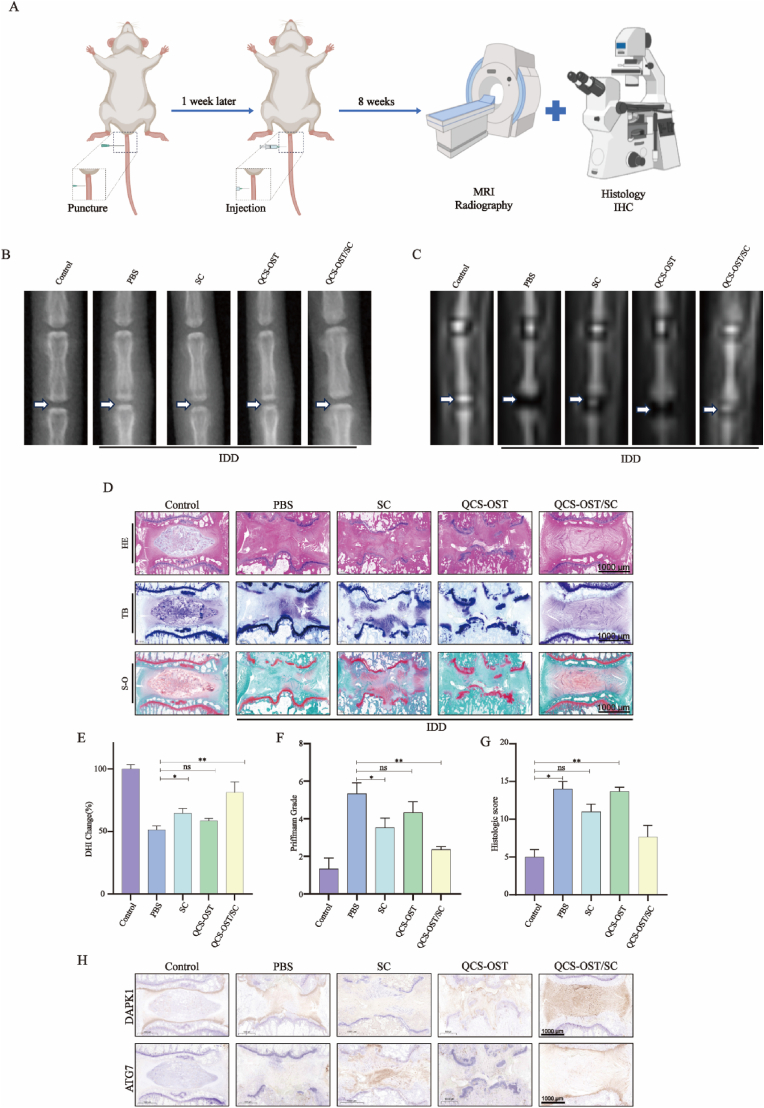
**Therapeutic effect of QCS-OST/SC in puncture-induced rat IDD model. (A)** Summarizes the design of the animal study protocol. **(B)** Presents a selection of X-ray images from various experimental groups at the 8-week mark. **(C)** Displays a collection of MRI images from different study groups captured at 8 weeks post-treatment. **(D)** Illustrates a series of histopathological staining (H&E, Toluidine Blue O, and Safranin O with Fast Green) from different groups at various time points (scale bar = 1000 μm). **(E)** Provides a numerical assessment of the disc height index (DHI) as derived from X-ray images, with a sample size of three individual discs per group. **(F)** Offers a comparative analysis of T2-weighted disc signal intensity measured using Image J software and normalized to the control group, with three individual discs per group. **(G)** Presents the histological scoring of the experimental groups at 8 weeks post-surgery, with a sample size of three individual discs per group. **(H)** Displays immunohistochemical (IHC) outcomes for DAPK1 and ATG7 proteins in the Control, IDD, SC, QCS-OST, and QCS-OST/SC groups after 9 weeks of in vivo treatment (scale bar = 1000 μm). The data in the figure represent mean ± S.D. Significant differences between the treatment and control groups are expressed as ∗∗p < 0.01, ∗p < 0.05,ns = no meaning,n = 3.

The effectiveness of OCS-OST/SC hydrogels was examined in rat models of needle-induced intervertebral disc degeneration (Fig. 8A). The IDD was assessed in rats using X-ray and MRI. SD rats were administered injections of 5 μL of PBS, SC, QCS-OST, or OCS-OST/SC via microneedle s into the intervertebral disc. The experimental group was acupunctured and the control group was not acupunctured with medicine. X-ray and MRI examinations at 8 weeks postpuncture revealed greater intervertebral height and stronger disc signals in the SC and QCS-OST/SC groups than in the IDD and QCS-OST groups (Fig. 8B and C). However, the efficacy of the QCS-OST/SC is better than that of the SC. It may be that it is difficult to ensure sustained injection of SC alone at high concentrations, which seriously affects the effectiveness of the drug. The DHI of the QCS-OST/SC group was significantly greater than that of the IDD, SC and QCS-OST groups, and the Pfirrmann score, a measure of disc degeneration, was notably lower in the QCS-OST/SC group than in the IDD, SC, and QCS-OST groups (Fig. 8E and F). Evaluation of tissue using HE revealed that QCS-OST/SC treatment delayed the degeneration of IDD tissue, preserving NP structures. The proteoglycan content in the NP was determined by Safranin O/Fast green staining. In the control setup, the NP area was densely packed with proteoglycans, as opposed to the IDD, QCS-OST, and SC groups where a substantial decrease in proteoglycan content was recorded. The group administered with QCS-OST/SC witnessed a less reduction in proteoglycans in comparison to the other groups that experienced puncture. Additionally, the nucleus of a mast cell was stained blue by toluidine blue, and there were fewer mast cells in the normal intervertebral disc. however, after intervertebral disc degeneration, the number of mast cells increased significantly (Fig. 8D). There were fewer mast cells in the QCS-OST/SC group. The expression levels of ATG7 and DAPK1 in the tissues were also assessed. IHC results showed that DAPK1 and ATG7 levels were significantly greater in the SC and QCS-OST/SC groups at week 8 than in the other needle groups, and these differences were further illustrated by semiquantitative staining analysis, suggesting that SC and QCS-OST/SC effectively promoted autophagy in the nucleus pulposus by increasing DAKP1 (Fig. 8H, Fig. S7). We confirmed this perspective through an in-depth evaluation of protein expression in multiple rat groups (Fig. S8). To ascertain the in vivo feasibility of our hydrogel, we assessed its biosafety in rats. H&E staining of the major organs, including the heart, liver, spleen, lungs, and kidneys, revealed no significant pathological alterations compared to the control group, thereby further confirming its favorable biosafety profile in vivo (Fig. S9). The hemolysis experiments images of RBCs also verified the excellent biocompatibility of QCS-OST/SC hydrogel (Fig. S10). The above results all indicate the effectiveness of SC in the treatment of IDD, but QCS-OST/SC can reduce the frequency of drug injection and significantly improve the efficacy and feasibility of SC treatment. These results were further supported by semiquantitative staining analysis, which provided valuable insights into the potential of QCS-OST/SC in enhancing autophagy in vivo for the treatment of IDD.

In summary, the therapeutic effect of SC on intervertebral discs involves promoting an increase in DAPK1 levels and further promoting autophagy. Despite the lack of significant impact from QCS-OST in both in vivo and in vitro experiments, it effectively resolves the problem of rapid SC clearance in vivo. Long-term injection of SC has been proven to cure intervertebral disc degeneration, demonstrating the effectiveness of SC. However, short-term, repeated disc injections can present difficulties, as even microneedle injections can cause some degree of disc damage. To address this issue, we synthesized a hydrogel by a Schiff base reaction between quaternary amino chitosan and oxidized starch. The hydrogel prolongs the duration of SC and is more effective against disc degeneration.

## 3 Disscussion

IDD is a widespread condition affecting the spine, typically associated with ongoing back discomfort and more commonly seen in the aging demographic [33]. The pathophysiology of IDD is intricately linked to the processes of apoptosis and cellular aging within the nucleus pulposus. Li's research underscores the influence of circRNAs on the regulation of gene transcription and translation, potentially impacting the survival of these cells and offering new avenues for IDD intervention [34]. Silwal's findings point to an increase in senescent cells in discs that have aged and degenerated, suggesting their role in the progression of IDD associated with aging [35]. Our investigation revealed that the exposure to TBHP led to a marked rise in both apoptosis and cellular senescence among nucleus pulposus cells, hinting at their involvement in the pathophysiological cascade of disc degeneration.

Autophagy, a cellular self-digestion mechanism, is essential for the maintenance of cellular integrity by eliminating obsolete proteins and damaged organelles [36]. A substantial amount of research has illustrated the protective role of autophagy in countering a spectrum of diseases, such as chronic obstructive pulmonary disease [37], osteoarthritis [38], and spinal cord injury [39]. The work of Chen and colleagues has indicated that the expression levels of genes and proteins involved in autophagy are significantly lower in degenerated intervertebral disc tissues compared to those in healthy tissues. Furthermore, their research has suggested that metformin has the potential to enhance autophagy in the nucleus pulposus cells, which could help in retarding the disc degeneration process [40]. The modulation of autophagy in intervertebral disc cells is being considered as a novel therapeutic strategy for the management of IDD. In this context, the identification of drugs that can stimulate autophagy is of considerable clinical importance for the treatment of disc degeneration. Our research introduces a novel finding that Spatholobi Caulis possesses the capacity to trigger autophagy in NPCs, presenting a potential therapeutic avenue for IDD.

In the context of IDD, the role of NPCs is pivotal. These cells are responsible for the synthesis of ECM, which is essential for maintaining the structural and functional stability of the disc. However, as IDD progresses, NP cells become compromised in their capacity to sustain this stability and their survival. Consequently, there is a progressive depletion of type II collagen and proteoglycans, leading to a reduction in the height of the IVD, a loss of the distinct boundary between the annulus fibrosus (AF) and the NP, and a diminished capacity to bear mechanical stress [41]. Key enzymes involved in the degradation of the NP matrix include matrix metalloproteinases (MMPs), integrins, and a disintegrin and metalloproteinase with thrombospondin motifs (ADAMTS). Notably, enzymes such as MMP-3, MMP-9, MMP-13, ADAMTS-4, and ADAMTS-5 are particularly implicated in the catabolic processes within the NP [42]. The upregulation of these metabolic enzymes in degenerated NP tissues indicates an imbalance between the catabolic and anabolic activities of NP cells. This imbalance, where the catabolism exceeds the protective anabolic functions, contributes to the development and progression of IDD.

In order to reveal the relationship between autophagy, apoptosis and senescence, we applied the autophagy inhibitor 3-MA in our study. We found that when the activation of autophagy was inhibited, the protective effect of SC on NPCs disappeared, suggesting that protective effect of SC on nucleus pulposus cells was mediated by autophagy stimulation.

Death-associated protein kinase 1 (DAPK1), a serine/threonine kinase regulated by calcium and calmodulin, is recognized for its significant involvement in numerous neuronal injury paradigms. Accumulating research suggests a correlation between genetic variations of DAPK1 and the onset of Alzheimer's disease [43]. However, DAPK1 has not been reported in intervertebral disc degeneration. Li, T. verified in a rat model that DAPK1 can ameliorate oxidative stress and inhibit autophagy by suppressing the p38 MAPK/NF-κB pathway [44], which contradicts the view of Singh, P. and others who suggest that DAPK1 promotes autophagy [45]. Therefore, there are some controversies about whether DAPK1 promotes or inhibits autophagy, but the increase in autophagy levels in NPCs treated with SC is an objective fact. Fig. 8 shows the potential of SC to delay disc degeneration by regulating NPCs' autophagy.

In recently year, a wide variety of materials and technologies have been developed for biomedical applications [[46], [47], [48], [49], [50]]. Hydrogels are a class of gels that use water as a solvent or dispersion medium. With properties such as reversibility, stimulus response, ductility, shape adaptability and rapid preparation, their soft nature and high water content provide a microenvironment similar to that of natural ECMs, and good biocompatibility is particularly suitable for cell growth [51]. Starch has been widely used in medicine, but it is limited by lack of ionizing functional group, weak adsorption and poor mechanical strength [52]. We modified it and greatly improved its defects. The high adsorption force can effectively adsorb SC. In order to further improve the performance of starch hydrogel, chitosan was modified into quaternary aminated chitosan and chemically cross-linked with oxidized starch by Schiff base reaction. A hydrogel with slow release (Fig. 6) and injectable (Fig. 5J) was designed and prepared. In addition, the reversible chemical bonds of hydrogels can maintain the integrity and mechanical properties of hydrogels, thereby extending their function and service life, that is, they have self-healing properties.

In the construction of intervertebral disc degeneration (IDD) models, there are various methods to choose from, such as needle puncture [53], chemical induction [54], and abnormal stress [55]. However, the needle puncture method has been widely used in a multitude of studies and possesses significant advantages. Firstly, the anatomical structure of the rat spine bears a certain similarity to humans, especially in the tissue structure of the intervertebral discs, which provides a foundation for simulating the pathological changes of human IDD. Secondly, by precisely controlling the parameters of needle puncture, such as the number of times, depth, and force, the key pathological processes of IDD can be replicated in the rat model, including the degeneration of the nucleus pulposus, the injury of the annulus fibrosus, and the reduction of disc height. These are important characteristics in the development of IDD. At the molecular level, the needle-induced model can reflect the biological molecular changes during the process of IDD, including the reduction of proteoglycans, the reorganization of collagen, and the release of inflammatory factors [[56], [57], [58], [59]]. These molecular events are key components of the pathological mechanisms of IDD. Therefore, we have chosen to utilize the needle puncture method for the construction of an intervertebral disc degeneration model for subsequent research.

Regarding the dose of hydrogel injected, current studies have different controversies. Barcellona et al. [60] proposed that the therapeutic dose of intervertebral disc injection in rats is 2 μL, while chen et al. [51] showed that an injection dose of 5 μL can obtain good results. In the experiment, we found that 5 μL of QCS-OST/SC hydrogel could achieve a good effect when injected, and no leakage problem was found.

Our research shows that the properties of modified hydrogels have been greatly improved. On one hand, compared to QCS hydrogel, QCS-OST hydrogel exhibits biocompatibility, superior rheological properties, self-healing capability, and injectability. QCS-OST hydrogel not only closely matches the mechanical properties required for intervertebral disc regeneration, effectively preventing mechanical stress imbalance and additional degenerative damage to the intervertebral disc and adjacent endplate cartilage, but also better prevents nerve compression that may occur after injection.

Despite these important findings, there are still some limitations to the study. First of all, the effect and mechanism of QCS-OST/SC hydrogel on NPCs was only discussed in this paper, but the intervertebral disc tissue and its surroundings contain T cells, B cells, neutrophils and endothelial cells, which participate in the progression of IDD by releasing cytokines and chemokines [61]. The effects of hydrogels on these cells need to be further explored in follow-up studies; Secondly, although the rat IDD model has been widely recognized, the disc degeneration in normal people is caused by a combination of many factors. Finally, the impact of microneedle injection on disc degeneration cannot be completely avoided, and it still needs to be further improved and perfected for clinical treatment, but it can provide a new idea for the current treatment of disc degeneration. On the other hand, both in vitro and in vivo experiments demonstrate that the QCS-OST hydrogel carrying SC (QCS-OST/SC hydrogel) not only ensures SC's effects in combating apoptosis and senescence and promoting ECM secretion in NPCs through autophagy, but also has advantages of prolonged, stable, safe, and efficient therapeutic effects. Additionally, QCS-OST/SC can counteract the significant increase in mast cells observed following intervertebral disc degeneration.

## 4 Conclusion

Our study revealed that the use of SC can improve the survival of NPCs by inhibiting apoptosis and senescence, a finding confirmed in experiments in IDD-induced SD rats using acupuncture. Nevertheless, the limited availability of SC when given orally or through microneedle injection into the intervertebral disc requires extended treatment periods. To combat this issue, we developed a QCS-OST/SC hydrogel with self-repair capabilities and injectable traits that displayed improved SC retention and regulated localized release. The QCS-OST/SC hydrogel effectively alleviated intervertebral disc degeneration triggered by acupuncture in rats by enhancing autophagy of NPCs, suppressing apoptosis and senescence, and altering abnormal ECM metabolism. These results highlight the potential of the innovative QCS-OST/SC hydrogel as a promising treatment option for IDD.

## 5 Methods

### 5.1 Reagents and antibodies

Spatholobi Caulis was acquired from Xi'an Xuhuang Biotechnology Co., Ltd., and subjected to water extraction by Xi'an Bioetec Biotechnology Co., Ltd. Chemical compounds such as 3-MA and TBHP were acquired from Sigma‒Aldrich in St. Louis, Missouri, USA. Various proteins, such as Beclin1, ATG7, Bcl-2, Cleaved-caspase3, BAX, P53, P21, P62, DAPK1, and GAPDH, were purchased from Wuhan Sanying Biotechnology Co., Ltd. Anti-Adamts5 was obtained from Boster Technology Co., Ltd., while anti-P16, Collagen II, MMP13, and Aggrecan were purchased from Abcam Technology Co., Ltd. Secondary antibodies were also obtained from Wuhan Sanying Biotechnology Co., Ltd. Furthermore, 4′,6-diamidino-2-phenylindole (DAPI) was obtained from Beyotime (Shanghai, China). The cell culture medium DMEM/F12 for NPCs was obtained from Gibco (Grand Island, NY, USA). Different kits, including a cytotoxicity and activity stain from Wuhan Cevive Biotechnology Co., Ltd., a FITC-Annexin V/PI apoptosis kit, and a β-galactosidase staining kit from Suzhou Youyilandi Biotechnology Co., Ltd., were utilized.

### 5.2 Nucleus pulposus cell culture

Forty young male Sprague‒Dawley rats weighing between 150 and 200 g each were humanely put down using an overdose of pentobarbital sodium. Under sterile conditions, the tail of each rat was carefully clipped, and the skin was removed to reveal the thick white ligament. The tail was then snipped with scissors to expose the white intervertebral disc tissue. Using a surgical blade, the clear nucleus pulposum tissue was removed. The harvested nucleus jejunal tissues were then placed in sterile PBS, spun in a centrifuge to eliminate the liquid on top, and treated with 0.1 % collagenase and 2 U/mL hyaluronidase at 37 °C for 4 h. Subsequently, the broken-down tissues were transferred as explants to a DMEM incubator supplemented with 10 % fetal bovine serum and antibiotics (1 % penicillin/streptomycin) at 5 % CO2 and 37 °C. After 1 week, the NPCs were separated from the medium and allowed to adhere. No notable changes in cell shape were observed between the original cells (generation 0) and subsequent cells (generation 2) when the cell density reached 80 %. For all experiments, the second set of cells was grown in a monolayer and maintained at 37 °C with 5 % CO2. The complete medium was replaced every 48 h.

### 5.3 Cell culture treatment plan

To establish the model of apoptosis (apoptosis) and senescence (senescence) in NPCs, various concentrations of TBHP (25, 50, 100, or 200 μM) were added to the cell culture and allowed to incubate for 24 h. Afterwards, the cells were treated with different concentrations of SC (12.5, 25, 50, or 100 μg/mL) for 24 h prior to the introduction of TBHP (50 μM) to examine its impact on apoptosis and senescence. To explore the involvement of autophagy in the protective effect of SC on cells, NPCs were treated with 10 μM 3-methyladenine (3-MA, a blocker of autophagy) for 1 h prior to SC administration. All trials were carried out in triplicate.

### 5.4 Preparation of QCS and OST

Conventional methods were used to prepare the QCS. First, 1 g of chitosan was dissolved in 72 mL of acetic acid solution (0.5 % v/v) and stirred continuously for 12 h. Afterwards, 2.82 g of GTMAC was added, and the mixture was stirred at 55 °C for 18 h. The resultant solution was encapsulated in a dialysis bag with a molecular weight cutoff of 10,000 Da and dialyzed in ultrapure water for 3 d. Ultimately, the solution was freeze-dried to produce purified QCS. Conversely, OST was synthesized using a standard approach involving the utilization of sodium periodate (NaIO_4_) as an oxidizing agent. Initially, soluble starch (1 g) was mixed with 55 mL of distilled water and heated to form a solution. Then, sodium periodate (NaIO_4_, 0.5 g) was introduced to this solution in a dark environment to initiate the oxidation process. The mixture was left to react at ambient temperature for a duration of 6 h, at which point glycol (0.1 mL) was added to terminate the reaction. Following this, the solution was dialyzed and freeze-dried to OST.

### 5.5 Synthesis of the QCS-OST and QCS-OST/SC hydrogels

A composite hydrogel integrating QSC and OST was synthesized through a Schiff base linkage. The formulation of QCS-OST involved the preparation of a 4 % w/v solution of quaternized chitosan in phosphate-buffered saline at a neutral pH of 7.4, to which an equivalent volume of 20 % w/v oxidized starch solution was added. To form the QCS-OST/SC hydrogel, 1 mg of SC was introduced into 4 mL of the precursor solution. The solution was then vigorously stirred on ice and incubated until the final hydrogel was obtained.

### 5.6 Physicochemical structure characterization

The molecular configurations of QCS and OST were elucidated through 400 MHz nuclear magnetic resonance (NMR) spectroscopy, facilitated by a Bruker spectrometer. For a comprehensive examination of the chemical compositions of chitosan, QCS, soluble starch (ST), OST, and the synthesized hydrogels, Fourier-transform infrared (FT-IR) spectroscopy was deployed, scanning across a wavenumber spectrum from 4000 to 500 cm^−1^ with a Thermo Scientific Nicolet 6700 analyzer.

### 5.7 Morphological characterization

The hydrogel specimens were sectioned into fine slices followed by freeze-drying. Subsequently, these samples were coated with a layer of gold and examined using a scanning electron microscope (SEM) model FEI FEG Quanta 250, operated at an accelerating voltage of 30 kV.

### 5.8 Rheology and self-healing properties of the hydrogels

A TA rheometer (DHR-2) was utilized to assess the rheological properties and self-repairing abilities of the hydrogels. The energy storage modulus, loss modulus, and critical strain of the hydrogel samples were tested. Furthermore, cutting and reattachment experiments were conducted to study the fusion mechanism and assess the self-healing capabilities of the hydrogels.

### 5.9 Release curve of SC in the QCS-OST/SC hydrogel

SC solutions were prepared at concentrations of 0.25, 0.5, 1.0, and 2.0 mg/mL using PBS. An ultraviolet spectrophotometer was utilized to measure the absorbance to establish the SC standard curve. Next, 100 μL of hydrogel and 1 mL of PBS (pH = 7.4) were added to a 4-well plate (Corning, 8 μm). The supernatant in the chamber was replaced with fresh PBS on days 0, 2, 4, 6, 8, and 14 for subsequent analysis. The quantity of SC released was then measured and computed, and the release pattern of the OCS-OST/SC hydrogel was graphed.

### 5.10 In vivo detection of QCS-OST/SC release

In vivo, Cy5-labeled CS was synthesized to assess the discharge of Cy5-QCS/SC and Cy5-QCS-OST/SC from the QCS-OST/SC hydrogels. A total of 10 adult male Sprague‒Dawley rats (3 months old) were randomly divided into two groups for testing. The first group was administered 5 μL of Cy5-QCS-OST/SC into the Co6/7 disk space using a 26-gauge needle, while the second group received a 5 μL solution of Cy5-QCS/SC at an equivalent concentration for comparison purposes. Monitoring of Cy5-SC (605/680 nm) placement and release was conducted using a Caliper IVIS Lumina II (CLS136341/F, USA). Imsenescence with the IVIS system was induced at 0, 3, 7, and 14 days after the injection, while the rats were anesthetized with 3 % (w/v) pentobarbital at a dose of 40 mg/kg. Quantification of the SC in the IVIS involved measuring the total radiation efficiency and normalization to day 0.

### 5.11 Cell viability assay

Cell viability was assessed by utilizing a cell counting kit 8 (CCK-8) from Sewell in Wuhan according to the manufacturer's instructions. Following a 24-h incubation period at 37 °C in DMEM supplemented with 10 % FBS, the second-generation nucleus pulposus cells were seeded onto 96-well plates at a density of 5000 cells per square centimeter. Subsequently, the cells were treated with TBHP, SC, and 3-MA. After treatment, the cells were washed with PBS, followed by incubation with 100 μL of CCK-8 solution in 10 mL of F12/DMEM (1:1) full medium for 2 h. The absorbance was then quantified at 450 nm using an enzyme labeler.

### 5.12 siRNA transfection

An siRNA duplex (Invitrogen) was custom-made and synthetically produced to target DAPK1 gene suppression in rat models. The specific sequences of the DAPK1 siRNAs used were as follows: sense strand, 5′-GGAATCTCTCACTGAAGAA-3’; 5′-GCCAAAGATTTCATCAGAA-3’; and 5′-GGATCCAATGCTATCTACT-3’. At the outset of transfection, the cells were plated in six-well plates and incubated for 24 h. Subsequently, 60–70 % confluent cells were transfected with either a control group or the DAPK1 siRNA duplex at a concentration of 100 nM, in accordance with the manufacturer's instructions, using the riboFECTTMCP transfection kit (RIBOBIO, Guangzhou) for a duration of 24 h. After transfection, the cells underwent additional treatments before being harvested for subsequent western blot analysis and immunofluorescence staining.

### 5.13 Transmission electron microscopy

After a 24-h treatment period, the cells from the nucleus pulposus were initially treated with 2.5 % glutaraldehyde overnight, fixed in 2 % osmium tetroxide for 1 h, and stained with 2 % uranedioxyacetate for an additional hour. Next, the specimens were dehydrated using a graded series of acetone concentrations and subsequently embedded in a resin using a cyclic vapor technique. The resulting embedded samples were cut into semithin slices, which were then stained with toluidine blue for cell identification. Ultimately, the sections were analyzed using transmission electron microscopy.

### 5.14 Western blot analysis

Total protein was extracted from the cells using Seville RIPA lysis buffer (Wuhan), and the protein concentration was measured utilizing a Beyotime BCA protein assay kit. Subsequently, the proteins were separated by SDS‒PAGE on polyvinylidene difluoride membranes (Millipore, USA). The membrane was initially blocked with 5 % skim milk and investigated with a variety of antibodies, such as against Cleaved-caspase3 (1:500), Bax (1:20000), Bcl-2 (1:3000), Beclin-1 (1:5000), P62 (1:50,000), ATG7 (1:5000), P16 (1:10,000), P53 (1:10,000), P21 (1:2000), GAPDH (1:10,000), Collagen II (1:1000), aggrecan (1:1000), ADAMTS5 (1:2000), MMP13 (1:1000), and DAPK1 (1:1000). After overnight incubation at 4 °C, the membrane was probed with secondary antibodies. Subsequently, the bands were detected using an electrochemical luminescent agent (procured from Suzhou Youyi Landi Biotechnology Co., Ltd.), and the band strength was quantified using Bio-Rad Image Lab 3.0 software.

### 5.15 Immunofluorescence

Pulpocytes from the nucleus were inserted into cell slides in six-well dishes, separated into the indicated drug treatment groups, fixed with 4 % paraformaldehyde, and placed in PBS supplemented with Triton X-100 for 10 min. After being incubated with 5 % bovine serum albumin for 30 min, the slides were incubated overnight at 4 °C with primary antibodies directed against aggrecan (1:500), ATG7 (1:500), Cleaved-caspase3 (1:400), or Collagen II (1:100). The slides were then rinsed and treated with a secondary antibody conjugated with either fluorescein isothiocyanate or tetramethylrhodamine isothiocyanate for 1 h and then stained with DAPI for 5 min. Microscopic analysis was conducted on random selected fields of view from each slide using a fluorescence microscope (Olympus Inc., Tokyo, Japan), and the intensity of fluorescence was assessed by a neutral observer using ImageJ software 3.0.

### 5.16 TUNEL assay

Terminal transferase nick-end labeling (TUNEL) is a crucial technique for quantifying DNA fragments resulting from apoptosis. Cells from the nucleus pulposus grown in culture for 12 h were seeded onto six-well plates. Following fixation with a solution of 4 % paraformaldehyde prepared fresh for a period of 60 min, the cells were treated with 3 % H_2_O_2_ and 0.1 % Triton X-100 for a duration of 10 min, with subsequent PBS washes performed after each step. Using an in situ apoptosis test kit from Suzhou Yuyi Lundi Biotechnology Co., Ltd., the cells were dyed with 4′,6-diamidino-2-phenylindole (DAPI). The identification and quantification of apoptotic changes were carried out using a fluorescence microscope from Olympus.

### 5.17 SA-β-gal staining

The process began with seeding cells into six-well plates, which were then rinsed twice with PBS. Subsequently, the cells were treated with a 0.2 % glutaraldehyde solution at room temperature for a duration of 10 min, followed by another PBS rinse and an overnight soak in an X-gal staining solution at a pH of 6.0. The staining solution contained specific concentrations of X-gal, citric acid/sodium phosphate, potassium ferricyanide, NaCl, and MgCl2. The cells' senescence was examined under an Olympus IX71 microscope at 20× magnification. A count of SA-β-gal-stained cells was performed on three randomly selected fields per group, and the proportion of positively stained cells was calculated for statistical analysis.

### 5.18 Surgical procedure

The rodents were weighed and received an intraperitoneal injection of 3 % (w/v) pentobarbital (40 mg/kg). Following procedures outlined in prior studies, the rat tail discs (Co6/7) were identified using manual palpation of the vertebrae of the tail and confirmed by counting vertebrae in the sacral area via X-ray imaging. A 27G needle punctured the entire annulus layer through the tail skin. To avoid excessive puncture, the length of the needle was calculated based on the measurements of the fibrous annulus and nucleus pulposus in the initial experiment, totaling approximately 4 mm. After the needles were left in the disc for 1 min, a mixture of SC and normal saline was prepared to achieve a final concentration of 500 μg/mL. Following the surgery, a solution containing 5 μL of SC, QCS-OST, or QCS-OST/SC was injected into the caudal vertebrae until the rats were euthanized. The rats' welfare was monitored daily, and the rats were permitted to move and carry weights without constraints.

### 5.19 X-ray and magnetic resonance imaging methods

After eight weeks, the animals were subjected to MRI. With the use of a 0.3 T magnetic resonance imaging device, MRI was performed on all the rodents, and both signal and structural modifications were assessed via sagittal T2-weighted images at the Nanchang Animal Institute Center. An additional orthopedic expert, unaware of the research, analyzed the MRI findings using the disc degeneration grading system established by Pfirrmann et al. (1 point = Grade I, 2 points = Grade II, 3 points = Grade III, 4 points = Grade IV, 5 points = Grade V).

### 5.20 Histopathological analysis

The rats were euthanized by administering an overdose of pentobarbital via intraperitoneal injection, followed by collection of their tails 8 weeks after the surgical procedure. The collected specimens underwent decalcification, fixation in formaldehyde, dehydration, and embedding in paraffin. Tissue sections with a thickness of 5 μm were then prepared. Slides of each disc were subsequently stained using HE, saffranine O-solid green (S-O), and toluidine blue. Experienced histological researchers blindly examined the cell structure and morphology of the nucleus pulposus and annulus fibrosus using a microscope. Evaluation was performed based on a grading scale previously reported in the literature. A histological score of 5 was considered normal, a score of 6–11 indicated moderate disc degeneration, and a score of 12–14 indicated severe disc degeneration.

### 5.21 Immunohistochemical examination

After the paraffin-embedded sections were dewaxed and rehydrated, they were subjected to microwave heating in a solution of 0.01 mol/L sodium citrate for 15 min. Following this step, the activity of endogenous peroxidase was blocked using 3 % hydrogen peroxide for 10 min at room temperature, and nonspecific binding sites were blocked with 5 % bovine serum albumin for 30 min. The next stage involved the incubation of the sections with the primary antibodies anti-ATG7 (1:500) and anti-DAPK1 (1:400) overnight at 4 °C. Next, the segments were treated with a suitable HRP-linked secondary antibody acquired from Santa Cruz Biotechnology (Dallas, TX, USA) and subsequently stained with hematoxylin. By utilizing Image-Pro Plus software version 6.0 from Media Cybernetics, Rockville, MD, USA, images were taken, and the total absorbance values were utilized as markers for the levels of ATG7 and DAPK1 expression. To evaluate protein expression, each sample was examined in at least three sections.

### 5.22 Hemolytic test

Rat-derived blood samples were initially collected and subjected to centrifugation at 1500 revolutions per minute (rpm) for a duration of 15 min. The erythrocytes were then suspended in sterile saline solution. A 100-μL aliquot of this erythrocyte suspension was combined with either 1.1 mL of saline solution for the negative control or 1.1 mL of deionized water for the positive control. Additionally, an equivalent volume of the erythrocyte suspension was mixed with 1.1 mL of hydrogel solutions containing varying concentrations of QCS-OST/SC to establish the experimental groups. Following this, all samples within Eppendorf tubes were incubated at a temperature of 37 °C within a water bath for a period of 1 h. Post-incubation, the supernatant was retrieved, and the absorbance at a wavelength of 540 nm was determined using an enzyme-linked immunosorbent assay (ELISA) reader. This data was utilized to ascertain the rate of hemolysis.

### 5.23 Statistical analysis

All the data are presented as the mean ± standard deviation (SD). Statistical analysis was performed using SPSS 20 software (IBM, USA). The normality of the parameters was assessed using the Kolmogorov‒Smirnov test. Group comparisons were conducted using two-tailed Student's t tests. Multiple comparisons were assessed using one-way analysis of variance (ANOVA) followed by post hoc Tukey tests. A P value < 0.05 was considered to indicate statistical significance.

## CRediT authorship contribution statement

**Shenghao Cai:** Writing – review & editing, Writing – original draft, Validation, Software, Data curation. **Rui Ding:** Writing – original draft, Software, Investigation. **Hongjun Zhang:** Writing – original draft, Software, Investigation. **Qirui Chen:** Writing – original draft, Visualization, Validation, Project administration, Methodology, Investigation. **Fen Yu:** Software, Resources, Formal analysis, Data curation. **Yong Xia:** Software, Resources, Investigation, Formal analysis, Data curation. **Qi Chen:** Software, Formal analysis, Data curation. **Xinxin Miao:** Writing – review & editing, Project administration, Methodology. **Bin Zhou:** Writing – review & editing, Software, Resources. **Jiahui Chen:** Supervision. **Le Liao:** Supervision. **Xigao Cheng:** Formal analysis, Data curation, Conceptualization. **Xiaoling Fu:** Investigation, Funding acquisition, Formal analysis, Data curation, Conceptualization.

## Ethics approval statement

This study was approved by the Ethics Committee of Nanchang University (Approval No.NCULAE-20221031080) and (Approval No.NCULAE-20221031118).

## Funding

The present study was supported by Key project of 10.13039/501100009102Jiangxi Provincial Department of Education (Grant No: GJJ210116).

## Declaration of competing interest

The authors declare the following financial interests/personal relationships which may be considered as potential competing interests: Xiaoling Fu reports financial support was provided by 10.13039/501100009102Jiangxi Provincial Department of Education. Xiaoling Fu reports a relationship with 10.13039/501100009102Jiangxi Provincial Department of Education that includes: funding grants. If there are other authors, they declare that they have no known competing financial interests or personal relationships that could have appeared to influence the work reported in this paper.

## Data Availability

The data that has been used is confidential.
