# Supplementary material for: Self-healing hydrogels loaded with Spatholobi Caulis alleviate disc degeneration by promoting autophagy in nucelus pulposus
Source: Mater Today Bio. 2024 Nov 8;29:101323. doi: 10.1016/j.mtbio.2024.101323 (PMC11609258; doi:10.1016/j.mtbio.2024.101323)
Supplement: Multimedia component 1 [file mmc1.docx]

**Self-healing hydrogels loaded with Spatholobi Caulis alleviate disc degeneration by promoting autophagy in nucelus pulposus**

**Shenghao Cai^1, 5✝^, Rui Ding^1, 5✝^, Hongjun Zhang^3✝^, Qirui Chen^1^, Fen Yu^4^, Yong Xia^1^, Qi Chen^1^, Xinxin Miao^1,2^, Bin Zhou^1^, Jiahui Chen^5^, Le Liao^5^, Xigao Cheng^1,2^*, Xiaoling Fu^1^***

^1^ Department of Orthopedics, The Second Affiliated Hospital, Jiangxi Medical College, Nanchang University, Nanchang, Jiangxi, China;

^2^ Institute of Orthopedics of Jiangxi Province, Nanchang, Jiangxi, China;

^3^ Affiliated Rehabilitation Hospital of Nanchang University;

^4^School of Materials Science and Engineering, East China Jiaotong University, Nanchang, Jiangxi, China;

^5^ Jiangxi Medical College, Nanchang University, Nanchang, Jiangxi, China;

*Correspondence: Xiaoling Fu, E -mail: [fxl1982@sina.com](mailto:fxl1982@sina.com); Xigao Cheng, E-mail:xigaocheng@hotmail.com

^†^ These authors have contributed equally to this work

*Correspondence:Xiaoling Fu,E -mail: fxl1982@sina.com;Xigao Cheng,E-mail:xigaocheng@hotmai l.com

^†^ These authors have contributed equally to this work


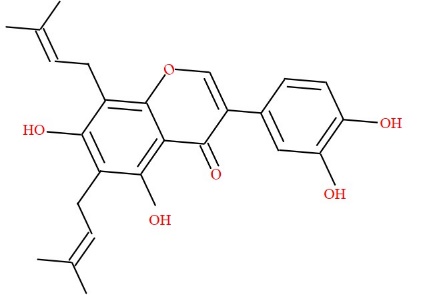


**Figure S1.** The chemical structure of Spatholobi Caulis (SC).


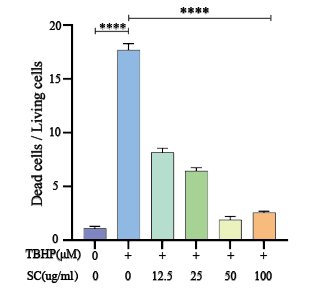


**Figure S2.** Qualification of protein bands shown in Figure 1C.


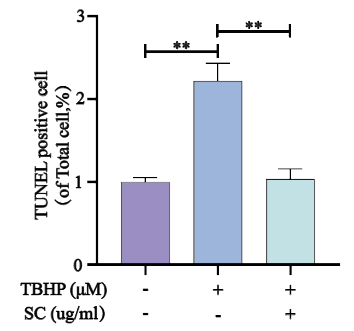


**Figure S3.** Qualification of protein bands shown in Figure 1H.


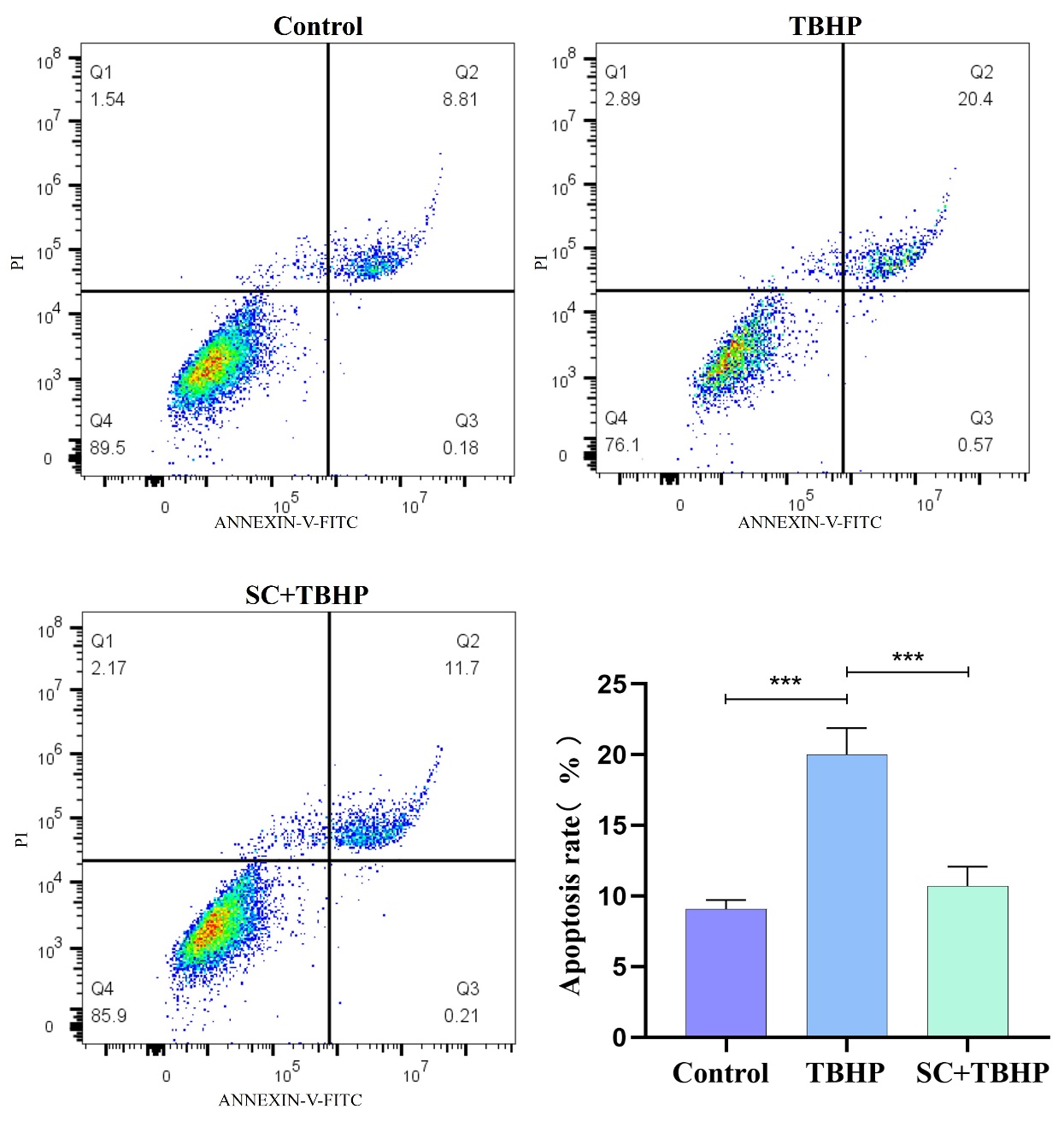


**Figure S4.** After TBHP and SC+TBHP (50ug/ml) treatment, apoptosis rate of NPCs (n = 3; ***p < 0.001 and NS = no meaning).


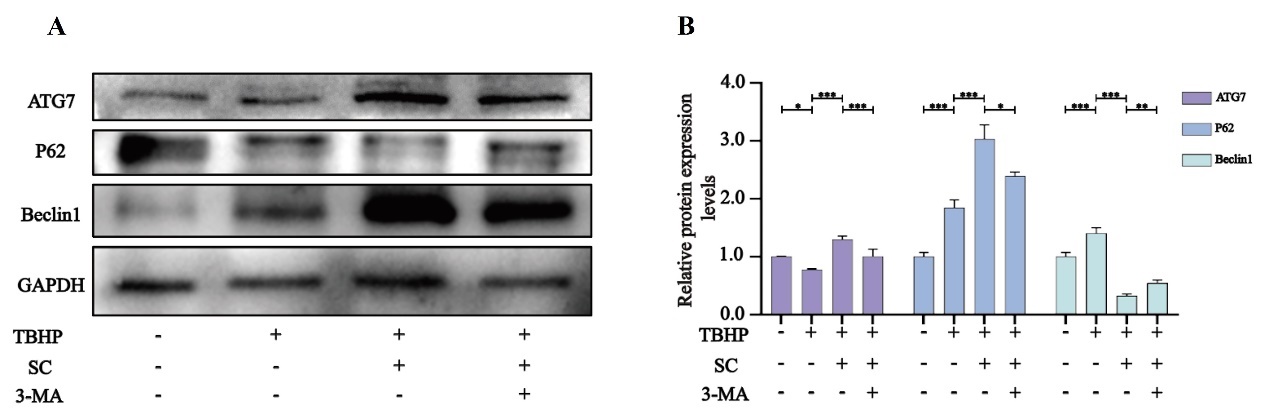


**Figure S5.** (**A**) Nucleus pulposus cells were untreated (DMEM 10% FBS), treated with TBHP (50 μΜ) alone, with SC (50 ug/ml) and TBHP, or with TBHP and SC (50 ug/ml ) combined with 3-MA (10 mM). Protein expression of ATG7, Beclin-1, and P62 in treated nucleus pulposus cells as described above. (**B**) Qualification of protein bands shown in Figure S2A. Data are presented as mean ± SD (n = 3). *P < 0.05, **P < 0.01 and ***P < 0.001.


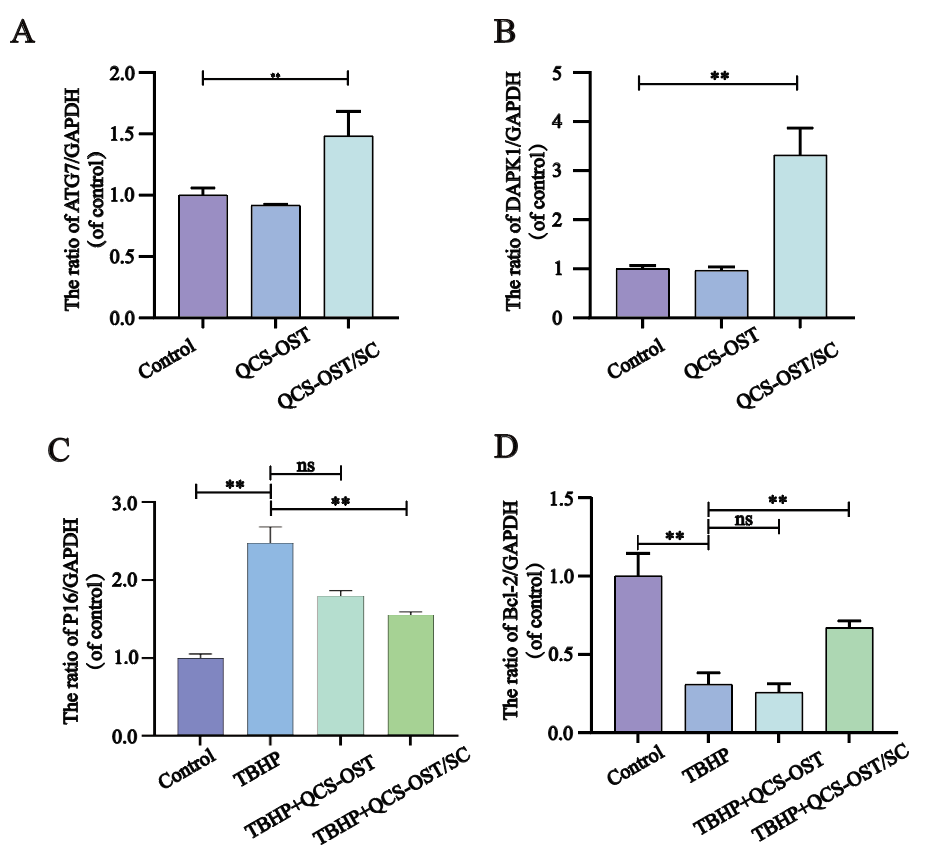


**Figure S6.** (**A-B**) Qualification of protein bands shown in Figure 6E. (**C-D**) Qualification of protein bands shown in Figure 6F. Statistical differences were determined using an ANOVA with Bonferroni's multiple comparison test (*p < 0.05, **p < 0.01).


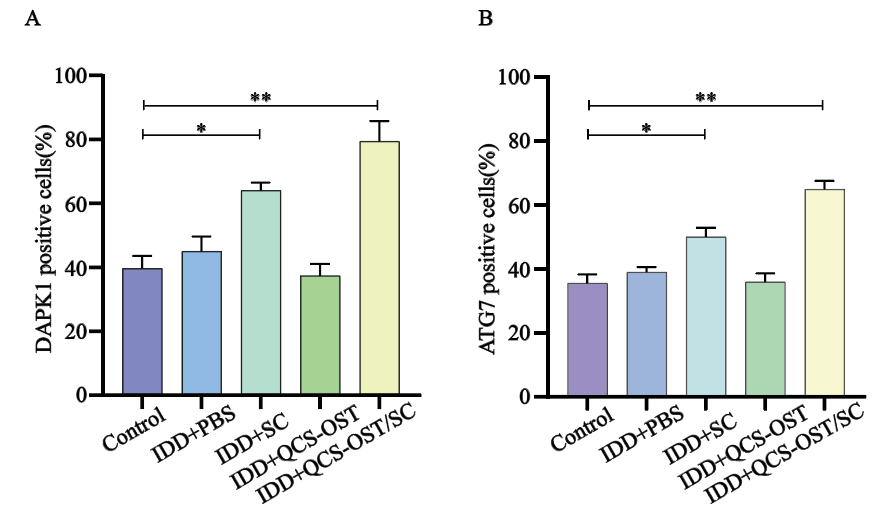


**Figure 7. (A-B)** Quantitative analysis of DAPK1 and ATG7 positive cells at the disc site (n=3). Statistical differences were determined using an ANOVA with Bonferroni's multiple comparison test (*p < 0.05, **p < 0.01).


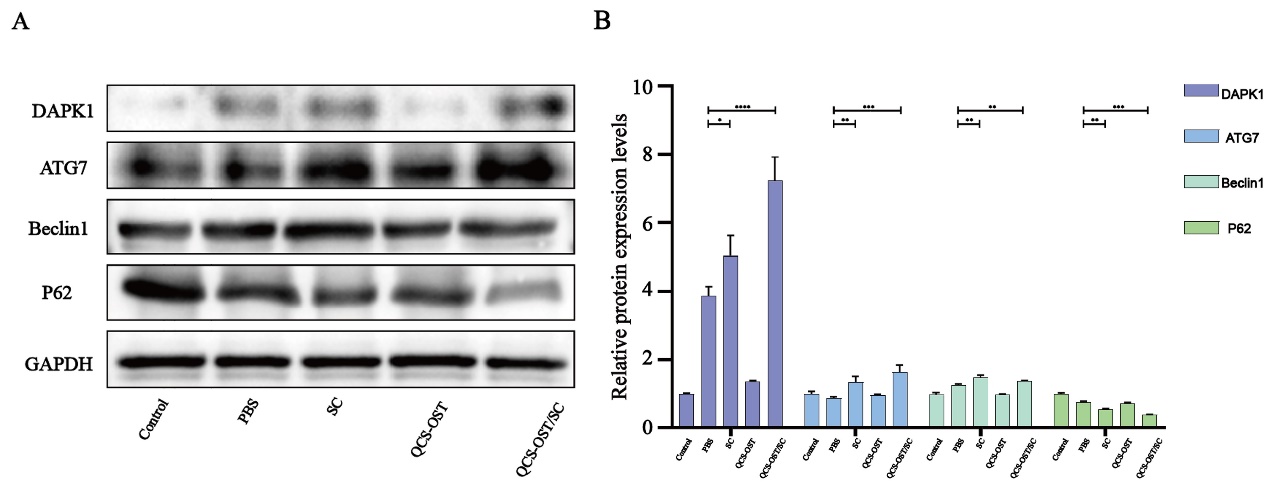


**Figure S8. (A)** Protein expression of ATG7, Beclin-1, and P62 in the Control, IDD, SC, QCS-OST, and QCS-OST/SC groups after 9 weeks of in vivo treatment. (**B**) Qualification of protein bands shown in Figure A Statistical differences were determined using an ANOVA with Bonferroni's multiple comparison test (*p < 0.05, **p < 0.01, ***p < 0.001,****p< 0.0001).


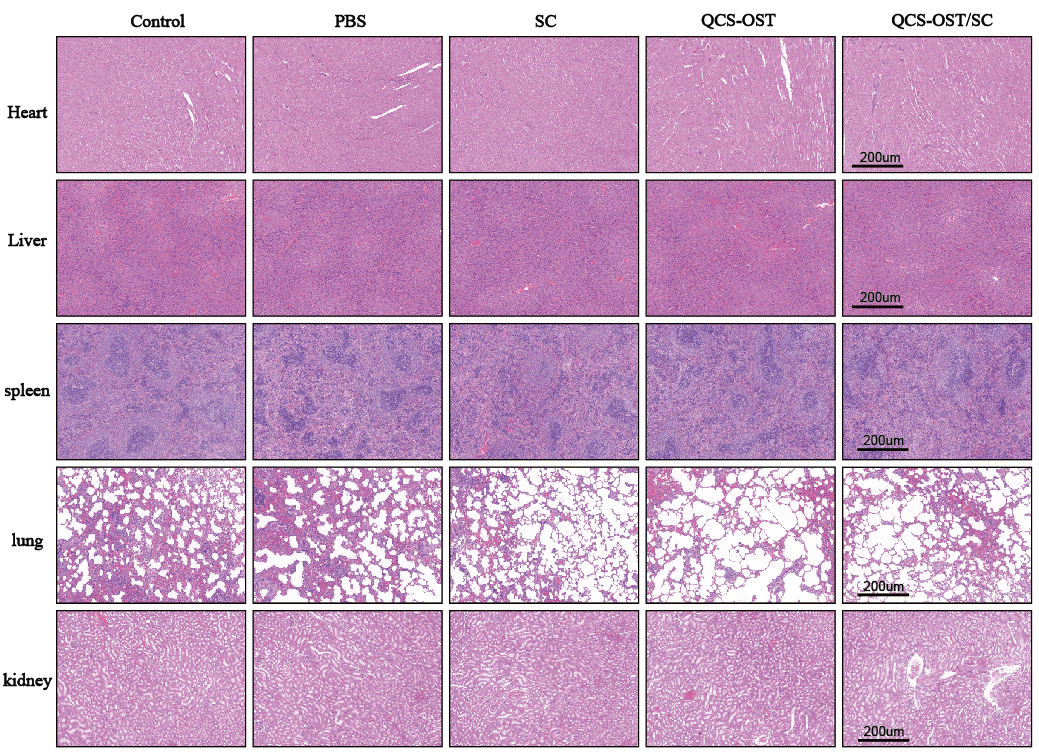


**Figure S9.** HE staining of heart, liver, spleen, lung and kidney of rats in different groups (scale bar = 200 μm).


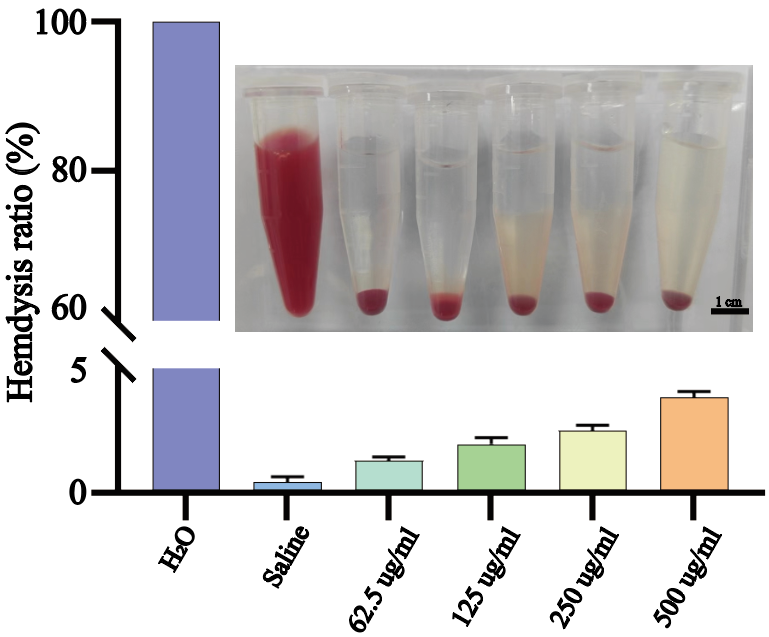


**Figure S10.** Hemolysis rate of different concentrations of QCS-OST/SC hydrogel. (Scale bar = 1 cm).
